# FTY720 Exerts Anti-Glioma Effects by Regulating the Glioma Microenvironment Through Increased CXCR4 Internalization by Glioma-Associated Microglia

**DOI:** 10.3389/fimmu.2020.00178

**Published:** 2020-03-04

**Authors:** Xu-Dong Guo, Juan Ji, Teng-Fei Xue, Yu-Qin Sun, Ruo-Bing Guo, Hong Cheng, Xiu-Lan Sun

**Affiliations:** ^1^Neuroprotective Drug Discovery Key Laboratory of Nanjing Medical University, Jiangsu Key Laboratory of Neurodegeneration, Nanjing Medical University, Nanjing, China; ^2^The First People's Hospital of Changzhou, Changzhou, China; ^3^The First Affiliated Hospital of Nanjing Medical University, Nanjing, China; ^4^Collaborative Innovation Center for Personalized Cancer Medicine, Center for Global Health, School of Public Health, Nanjing Medical University, Nanjing, China

**Keywords:** FTY720, glioma microenvironment, glioma-associated microglia and macrophages, chemoattraction, polarization

## Abstract

**Background:** Glioblastoma (GBM) is one of the most malignant and aggressive primary brain tumors. The incurability of glioblastoma is heavily influenced by the glioma microenvironment. FTY720, a potent immunosuppressant, has been reported to exert anti-tumor effects in glioblastoma. However, the impact of FTY720 on the glioma microenvironment remains unclear.

**Methods:** We examined the effects of FTY720 on the distribution and polarization of glioma-associated microglia and macrophages (GAMs) in glioma-bearing rats using immunofluorescence staining. qRT-PCR and Western blotting were used to detect the expressions of CXCR4 and MAPK pathway-related signal molecules on microglia in the coculture system. The levels of inflammatory factors were tested via ELISA. Wound healing assay and Matrigel invasion assay were used to determine the migration and invasion of C6 glioma cells.

**Results:** We discovered that FTY720 could inhibit the growth, migration, and invasion of glioma by targeting GAMs to impede their effect on glioma cells. Simultaneously, FTY720 could block the chemoattraction of GAMs by inhibiting MAPK-mediated secretion of IL-6 through increased internalization of CXCR4. Moreover, microglia and macrophages are polarized from pro-glioma to an anti-tumor phenotype.

**Conclusion:** These results provide novel insights into the inhibitory effects of FTY720 on glioma by targeting GAMs–glioma interaction in the tumor microenvironment.

## Background

Glioblastoma, characterized by mortality, is aggressive and invasive, and accounts for up to 50% of primary malignant brain tumors. Despite recent advances in therapy, including mass surgical resection, radiotherapy, and chemotherapy, patient prognosis remains poor (1–3). The 2-year survival rate is <5%, and the median overall survival is only 14 months (4, 5). Therefore, there is an urgent clinical need for new and effective treatment strategies for GBM patients.

While most therapeutic strategies have traditionally focused on curbing cancer cell proliferation and invasion, accumulating data suggest that the glioma microenvironment plays an integral role in the development and progression of glioma (6–8). Therefore, comprehensive therapeutic strategies should include targeting microenvironmental components, such as microglia, macrophages, astrocytes, and endothelial cells. Microglia are resident immune cells of the brain and, together with macrophages, account for ~30–50% of the infiltrative cells in gliomas, indicating a key role in regulating the glioma microenvironment (9–11). Glioma-associated microglia and macrophages (GAMs) are attracted to tumor cells to promote tumor cell proliferation and invasion, modify the extracellular matrix, and induce an immunosuppressive environment (12).

Studies have indicated that GAMs are recruited to the glioma microenvironment and release various growth factors and cytokines, which, in turn, facilitate tumor cell proliferation, migration, and invasion (13). GAMs-attracting chemokines and cytokines secreted by glioma cells, which suppress immune cells, include CCL2, CXCL12, CSF-1, and MIF. GAMs become immunosuppressive as a result of aberrations in the expression of corresponding receptors, including CCR2, CXCR4, CSF-1R, and CD74 (14). One study illustrated that glioma-derived CCL2 acts upon CCR2-bearing microglia to produce IL-6, which then stimulates gliomas (15). Ghoochani et al. demonstrated that interference with MIF–CD74 signaling in microglia represents a viable therapeutic option for the restoration of IFN-γ-driven immune surveillance (16). In that, blocking the chemoattractant receptors or ligands may elicit anti-glioma responses (17). Studies have also indicated that an immunosuppressive glioma microenvironment can push microglia and macrophages into an immune-paralyzing and pro-tumorigenic phenotype (18–20). Therefore, a potential therapeutic strategy for glioma is transforming GAMs into anti-glioma phenotypes.

FTY720, an immunomodulator approved for the treatment of multiple sclerosis by the Food and Drug Administration (FDA), has been shown to be therapeutically active in multiple cancers, including glioma (18, 21–23). However, mechanisms involved in FTY720-mediated anti-glioma activity remain unclear. In this study, we focused on the effects of FTY720 on regulating the cross-talk between chemoattractant receptor-bearing GAMs and ligand-secreting gliomas in the glioma microenvironment.

We established the C6 and 9L glioma model in rats and C6-microglia co-culture system to investigate the cross-talk between glioma and GAMs regulated by FTY720. We observed that FTY720 could increase CXCR4 internalization, and thereby reduce the receptor CXCR4 levels on the surface of microglia, leading to the inhibition of MAPK-mediated IL-6 release in the tumor microenvironment. Additionally, we discovered that FTY720 could transform GAMs into an anti-tumorigenic phenotype. These findings offer novel evidence for the potential clinical use of FTY720 as a therapeutic strategy for patients with glioma.

## Materials and Methods

### Animals

Six-week-old male Wistar rats weighing 250 ± 10 g were purchased from Beijing Vital River Laboratory Animal Technology Company. All rats were kept in a standardized environment (temperature 25 ± 2°C, humidity 55 ± 10%, irradiation time 8:00–20:00 h) in the Animal Resource Centre of the Faculty of Medicine, Nanjing Medical University. The entire experimental process and animal treatment adhered to the rules of the Experimental Animal Application Criteria and Institutional Animal Care and Use Committee (IACUC).

### Allograft Models and Treatment

Tumors were implanted into the right caudate nucleus of Wistar rats. The rats were anesthetized and placed in a stereotaxic apparatus. C6 and 9L glioma cells (1 × 10^6^) were injected subcutaneously into the right caudate nucleus of the rats (1 mm anterior, 3 mm lateral to the bregma, depth 5 mm from dura). FTY720 (2 mg/kg) or saline was intraperitoneally injected into models daily beginning on day 2 after tumor implantation. MRI was used to examine the tumor growth 14 days after implantation.

### Cell Culture

Rat primary microglial cells were generated from 1-day-old postnatal Sprague–Dawley rats as described previously (24). Briefly, microglia were isolated from cerebral cortices by mechanical dissociation, 0.25% trypsin/EDTA (Gibco, Grand Island, NY, USA), and plated into poly-D-lysine-coated (0.1 mg/ml; Sigma Chemical, St. Louis, MO, USA) T25 culture flasks. The rat glioma cell line C6 was cultured in Dulbecco's Modified Eagle's Medium (DMEM) (Gibco) containing 10% fetal bovine serum (FBS; Gibco) in a humidified 5% CO_2_ and 37°C incubator. Co-culture experiments were performed as shown below.

### Reagents and Antibodies

Fingolimod (FTY720) HCl was purchased from Selleckchem (Houston, TX, USA) and anti-Iba1 antibody was purchased from FUJI FILM Wako Pure Chemicals (Japan). Anti-iNOS antibody was purchased from Santa Cruz Biotechnology, while anti-Mannose Receptor (CD206) antibodies were purchased from Abcam. Phospho-p44/42 MAPK (Erk1/2) (Thr202/Tyr204), p44/42 MAPK (Erk1/2), Phospho-MEK1/2 (Ser217/221), MEK1/2, Phospho-JNK (Thr183/Tyr185), JNK, Phospho-p38MAPK (Thr180/Tyr182), p38 MAPK, and Na/K-ATPase antibodies were purchased from Cell Signaling Technology. The S1PR1 and S1PR3 antagonist VPC 23019 was purchased from Avanti Polar Lipids (Alabaster, USA).

### RNA Extraction and Reverse Transcription

Total RNA was extracted from cultured cells using TRIzol reagent (YIFEIXUE BIOTECH) according to the manufacturer's instructions, and a NanoDrop 2000 spectrophotometer (NanoDrop Technologies, Thermo Scientific, USA) was used to measure the purity and concentration of the total RNA. cDNA was synthesized with a PrimeScript™ RT Master Mix (Takara, Japan) according to the manufacturer's protocol.

### Real-Time Quantitative PCR

Real-time quantitative PCR was performed using the SYBR Green mixture (Selleckchem, USA) in a QuantStudio 5 Real-Time PCR System (Applied Biosystems, USA) according to the manufacturer's method. GAPDH was used as an endogenous control and all data were assessed using the 2^−ΔΔ*CT*^ method.

### Western Blot Analysis

For Western blotting, cells were washed with phosphate-buffered saline and lysed in RIPA buffer with protease and phosphatase inhibitors (Sigma) and centrifuged at 14,000 × g at 4°C for 15 min. Cell membrane proteins were extracted using a Cell Membrane Protein and Cytoplasm Protein Extraction Kit (KeyGEN BioTECH, China) according to the manufacturer's protocols, and the concentration of each protein sample was measured using an enhanced BCA Protein Assay Kit (KeyGEN BioTECH, China). The proteins were resolved by SDS-PAGE and transferred onto PVDF membranes, which were then blocked in 10 mM Tris buffer with 5% skim milk and incubated with specific primary antibodies at 4°C overnight. After being washed, the membranes were probed with HRP-conjugated secondary antibodies, visualized by ECL, and measured with ImageJ software. All Western blots were performed at least three times.

### Immunofluorescence Staining and Histopathology

Brain sections and cells were immunofluorescently stained as previously described (19). Briefly, brain samples and cells were fixed using 4% paraformaldehyde for 24 h or 15 min, respectively. Brains were also embedded in wax and cut into 5-μm-thick sections. The samples were then blocked with 10% normal donkey serum and 0.01% Triton X-100 in PBS for 60 min at room temperature and incubated with primary antibodies at 4°C overnight. Sections were subsequently incubated with corresponding Alexa Fluor 488-, 546-, and 555-conjugated specific secondary antibodies (Invitrogen, USA). Cell nuclei were stained with Hoechst 33258. The sections and cells were scanned with a fluorescence microscope (Olympus, Japan) by one investigator, and the staining was quantified by two independent investigators.

For histopathology, brain sections were first incubated with hematoxylin for 15 min, washed with water for 5 min, and then flushed with 1% HCl four times and then washed for 20 min. Finally, the sections were stained with eosin and photographed.

### Magnetic Resonance Imaging

MRI was performed using Biospec 70/20 USR (Bruker, Germany) with 1H/19F circular polarized small volume coil for rat head. MSME pulse sequence (TR = 3 s and TE = 33 ms) was used to acquire multi-echo images [a field of view (FOV) 3.5 cm^2^, data matrix = 256 × 256 × 25 slices, thickness = 1 mm].

### Enzyme-Linked Immunosorbent Assay

Cell culture supernatants were collected and centrifuged for 20 min at 1,000 × g. The concentration of CXCL12, IL-6, TNF-α, and IFN-γ in the cell culture supernatants was detected according to the manufacturer's instructions.

### Wound Healing Assay

A wound healing assay was used to examine the cell motility of C6 glioma cells. C6 glioma cells were seeded in a 24-well plate. After 12 h, a pipette tip was used to scratch the center of the well. The cells were then treated with culture medium with different concentrations of FTY720 and photographed at 0, 3, 6, 12, and 24 h.

### Matrigel Invasion Assay

The Transwell insert was precoated with Matrigel matrix (Corning Inc., NY, USA), and incubated at 37°C for 1 h to solidify. The insert was hydrated with 200 μl of DMEM and then 1 × 10^4^ C6 glioma cells in 200 μl of DMEM were seeded in the insert. The lower chamber was filled with 600 μl of DMEM containing 10% FBS with/without 5 × 10^4^ microglial cells to chemoattract C6 cells. After 24 h, the insert was washed twice with PBS, fixed with 5% Glutaral, and stained with 0.1% crystal violet (Sigma). A wet cotton swab was used to gently remove the cells on the top of the insert, and the cells were counted in four independent microscopic fields.

### Statistical Analysis

GraphPad Prism 7.0 was used for statistical analysis. Comparisons among groups were performed with one-way ANOVA, and unpaired Student's *t*-test was conducted between two groups. Data are expressed as mean ± SEM, and *p* < 0.05 was considered statistically significant.

## Results

### FTY720 Exerts Anti-glioma Effects in C6 and 9L Glioma Allograft Model

**Figure 1:**
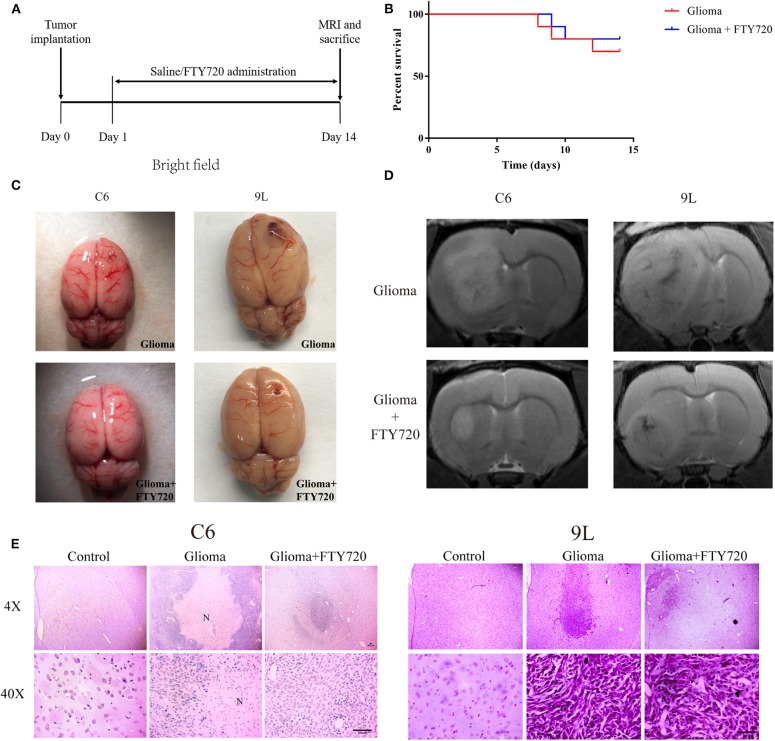
The anti-tumor effects of FTY720 on C6 and 9Lglioma rat models. **(A)** Experimental progress and treatment of C6 and 9L glioma rat models. **(B)** Survival differences of glioma rat models treated with saline or FTY720 for 14 days. *n* = 10 for each group. **(C)** Development of glioma in C6 and 9L glioma allograft models. **(D)** Representative MRI analysis of gliomas treated with saline or FTY720 14 days after tumor implantation. **(E)** Histopathological analysis of tumor distributions in brain sections. Upper and lower panels show lower (4×) and higher (40×) magnifications, respectively. N, necrotic area. Scale bar, 300 μm, *n* = 5–6.

Previous studies have shown that FTY720 possesses potent inhibitory effects in numerous cancer models, including breast cancer, multiple myeloma, and glioblastoma. In our study, we used C6 and 9L glioma allograft to evaluate the anti-tumor effects of FTY720. We found that treatment with FTY720 for 14 days did not alter the survival rate in C6 glioma-bearing Wistar rats (Figures 1A,B), while tumor volume was significantly reduced (Figure 1C), detected by MRI (Figure 1D) and H&E staining (Figure 1E), in contrast to counterparts treated with saline. Our results demonstrated the anti-glioma effect of FTY720 in C6 or 9L glioma-bearing rats.

### FTY720 Blocks the Chemoattraction of GAMs via Internalizing CXCR4

**Figure 2:**
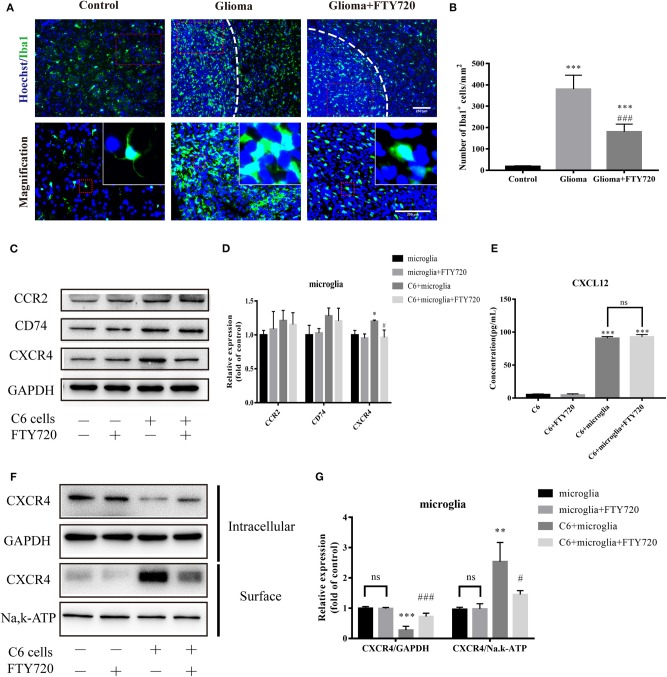
FTY720 blocks the chemoattraction of GAMs via internalizing CXCR4. **(A,B)** Representative immunostaining and analysis of Iba1-positive cells in the brain sections. Upper and lower panels show lower (20×) and higher (40×) magnifications, respectively. Data are presented as mean ± SEMs, *n* = 5–6. ***p < 0.001 vs. Control, ###*p* < 0.001 vs. Glioma. **(C,D)** Quantitative Western blotting analysis of the whole protein levels of CCR2, CD74, and CXCR4. **(E)** The concentration of CXCL12 in the cell culture supernatants. **(F,G)** The intracellular and cell-surface CXCR4 is analyzed by Western blots. Data are presented as mean ± SEMs, *n* = 3 for WB and *n* = 4 for ELISA assays. *p < 0.05 vs. microglia, **p < 0.01, ***p < 0.001 vs. microglia, ^#^*p* < 0.05, ^*###*^*p* < 0.001 vs. C6 + microglia.

Enormous inflammatory infiltrates, predominated by microglia and macrophages, are considered to be attracted, recruited, and subverted by glioblastoma cells for tumor growth (17, 20, 25). Therefore, we evaluated the recruitment and accumulation of GAMs in the glioma microenvironment with and without FTY720 treatment. Iba1^+^ GAMs numbers and cellular size increased and even enwrapped gliomas in C6 glioma-bearing rats, which were alleviated by FTY720 administration (Figures 2A,B). These results suggest that FTY720 could block the recruitment of GAMs.

To investigate how FTY720 mediates these changes in GAMs, we focused on various receptors, which may play significant roles in GAMs recruitment and chemoattraction. To do this, we established a C6-microglia co-culture system *in vitro* (**Figure 5D**), where we determined the expression level of different receptors expressed on the microglia. Among the chemokine receptors induced upon C6-microglia co-culture, we found specific upregulation of CCR2, CD74, and CXCR4. After treatment with FTY720, the expression of microglial CXCR4 was significantly reduced (Figures 2C,D). We then performed ELISA analysis to determine the changes of glioma-secreting chemokine responding to the reductions of microglial CXCR4. The ELISA results showed that FTY720 did not affect CXCL12 secretion by glioma cells (Figure 2E), indicating that FTY720 could regulate CXCL12–CXCR4 cross-talk between microglia and glioma via decreasing the expressions of CXCR4 on the microglia. Beider et al. demonstrated that CXCR4 can be directly targeted by FTY720, thus limiting tumor-promoting activities in multiple myeloma (26). Similarly, we found that FTY720 reduced cell-surface levels and increased intracellular levels of CXCR4. This indicates that FTY720 can also promote CXCR4 internalization by microglia (Figures 2F,G), thereby blocking the chemoattraction of GAMs.

### FTY720 Suppresses Glioma Migration and Invasion by Inhibiting the Activation of Mitogen-Activated Protein Kinase (MAPK) Signaling Pathway of GAMs

**Figure 3:**
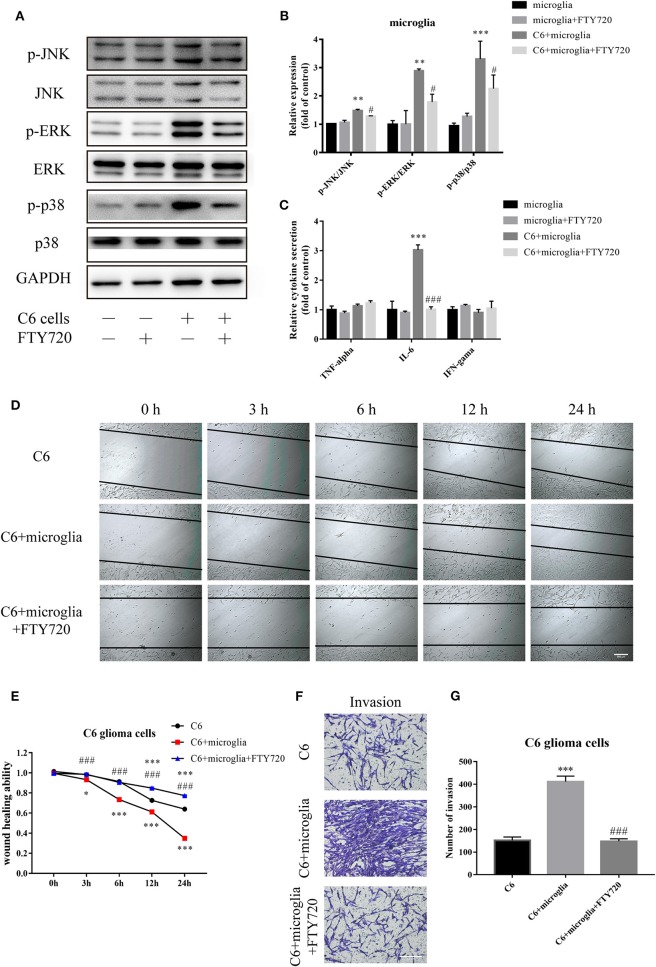
FTY720 decreases microglial MAPK-mediated IL-6 secretion to suppress the migration and invasion of C6 cells. **(A,B)** Western blots analyze the phosphorylation of p38, JNK, and ERK1/2. **(C)** The concentration of TNF-α, IL-6, and IFN-γ in the cell culture supernatants. **(D,E)** Migration of C6 cells at different times (0, 3, 6, 12, and 24 h) with/without FTY720 and representative analysis of C6 cell motility. **(F,G)** Representative images and analysis of C6 cell invasion under different treatment. Data are presented as mean ± SEMs, *n* = 4 for ELISA assays, **p < 0.01 vs. microglia, ^#^*p* < 0.05 vs. C6 + microglia; *n* = 3 for wound healing assays and Matrigel invasion assays, *p < 0.05, ***p < 0.001 vs. C6, ^*###*^*p* < 0.001 vs. C6 + microglia.

CXCR4 receptor activation is mediated by a GPCR mechanism, coupling to an intracellular heterotrimeric G-protein associated with the inner surface of the plasma membrane (27). CXCR4 signaling is rapidly desensitized after ligand binding by receptor internalization. To explore whether the downstream pathways of CXCR4 are indeed involved in mediating the effects of FTY720, we next assessed changes to CXCR4-regulated signaling pathways. CXCR4 signaling has been shown to be involved in the MAPK signaling pathway in cancer, mediating functions that include cell migration and survival (27). Our observations suggested that FTY720 could inhibit the activation of CXCR4-dependent intracellular MAPK signaling, as there was a reduction in the phosphorylation of three members of the MAPK pathway: ERK, JNK, and p38 (Figures 3A,B).

MAPK signaling was found to play vital roles in the release of chemokines and inflammatory cytokines, which in turn sustain tumor growth, invasion, and tumor escape (14). Therefore, we tested whether microglial MAPK signaling inhibited by FTY720 could influence the secretion and release of common related cytokines and chemokines, including TNF-α, IL-6, and IFN-γ. Our analysis showed a significant decrease in the expression level of IL-6 (Figure 3C). Studies revealed that IL-6 can contribute to the proliferative and migratory abilities of glioblastoma (28). Furthermore, we found that FTY720 inhibited the migration (Figures 3D,E) and invasion (Figures 3F,G) of C6 in a glioma–microglia co-culture system. Altogether, FTY720 decreased microglial MAPK-mediated IL-6 secretion to suppress the migration and invasion of glioma.

### FTY720 Exerts Anti-glioma Effects via CXCR4

**Figure 4:**
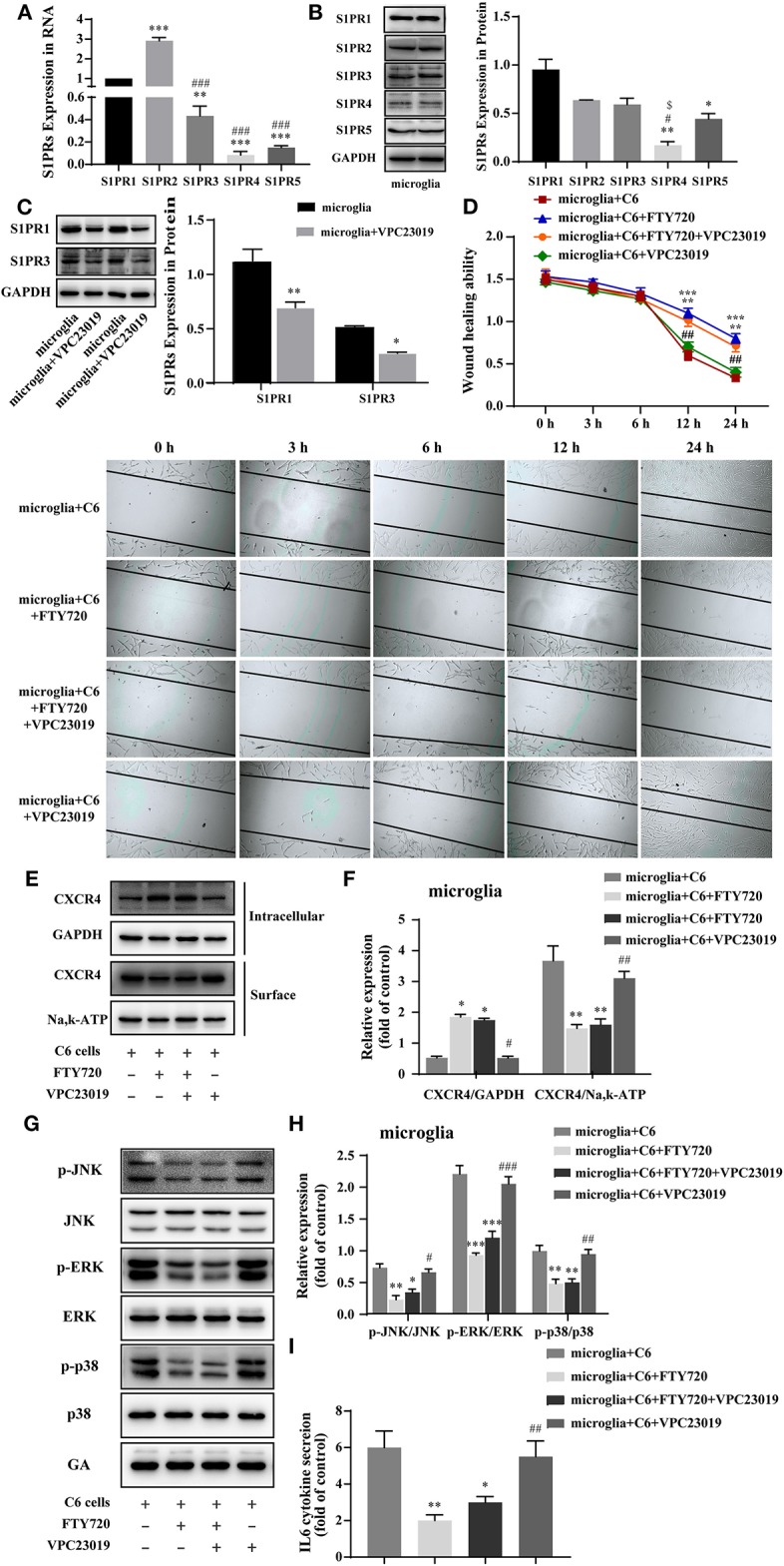
FTY720 exerts anti-glioma effects via CXCR4 instead of S1PRs. **(A,B)** The representative RNA and protein expression of S1PR1, S1PR2, S1PR3, S1PR4, and S1PR5 in microglia. **(C)** Western blots analyzed the expression of S1PR1 and S1PR3 in microglia treated with VPC 23019, respectively. **(D)** Representative images and analysis of C6 cells motility under different treatment. **(E,F)** The intracellular and cell-surface CXCR4 under different treatment is analyzed by Western blots. **(G,H)** Western blots analyze the phosphorylation of p38, JNK, and ERK1/2 under different treatments. **(I)** The concentration of IL-6 in the cell culture supernatants. Data are presented as mean ± SEMs, *n* = 3 for qPCR assays, **p < 0.01, ***p < 0.001 vs. S1PR1, ###*p* < 0.01 vs. S1PR2; *n* = 3 for Western blots of S1PRs, *p < 0.05, **p < 0. 01 vs. S1PR1, #*p* < 0.05 vs. S1PR2, *$p* < 0.05 vs. S1PR3; *n* = 3 for wound healing assays, **p < 0.01, ***p < 0.001 vs. microglia + C6, ##*p* < 0.01 vs. microglia + C6 + FTY720; *n* = 3 for Western blots, *p < 0.05,**p < 0.01, ***p < 0.001 vs. microglia + C6, ##*p* < 0.01, ###*p* < 0.01 vs. microglia + C6 + FTY720.

FTY720 is known as an immunomodulator and can be phosphorylated to be a sphingosine-1-phosphate (S1P) analog *in vivo* and binds to all of the sphingosine-1-phosphate receptors (S1PRs) (29, 30). To examine whether S1PRs play a role in the anti-glioma effects of FTY720, we first evaluated the expressions of S1PRs in microglia. The mRNA and protein expressions of S1PR1, S1PR2, and S1PR3 were relatively high in microglia (Figures 4A,B). Then, VPC 23019, a S1PR1 and S1PR3 antagonist (Figure 4C), was used to verify the roles of S1PRs on the anti-glioma effects of FTY720. We found that VPC 23019 failed to affect the motility of C6 glioma cells treated with FTY720 in glioma–microglia co-culture system (Figure 4D). Furthermore, we examined the effects of VPC 23019 and FTY720 on the internalization of CXCR4, downstream MAPK signaling, and IL-6 secretion. The results also showed that VPC 23019 did not affect the effects of FTY720 (Figures 4E–I). Altogether, our observations indicate that FTY720 exerts anti-glioma effects independent of S1PRs.

### FTY720 Polarizes GAMs From Pro-glioma to Anti-glioma Phenotypes

Glioma-derived IL-6, along with other cytokines such as TGF-β, polarizes glioma-infiltrating microglia and macrophages toward a pro-tumorigenic phenotype, which, in turn, produce and secrete IL-6 (13). Therefore, we tested whether FTY720 can influence GAM phenotypes and thus offer clinical benefits.

**Figure 5:**
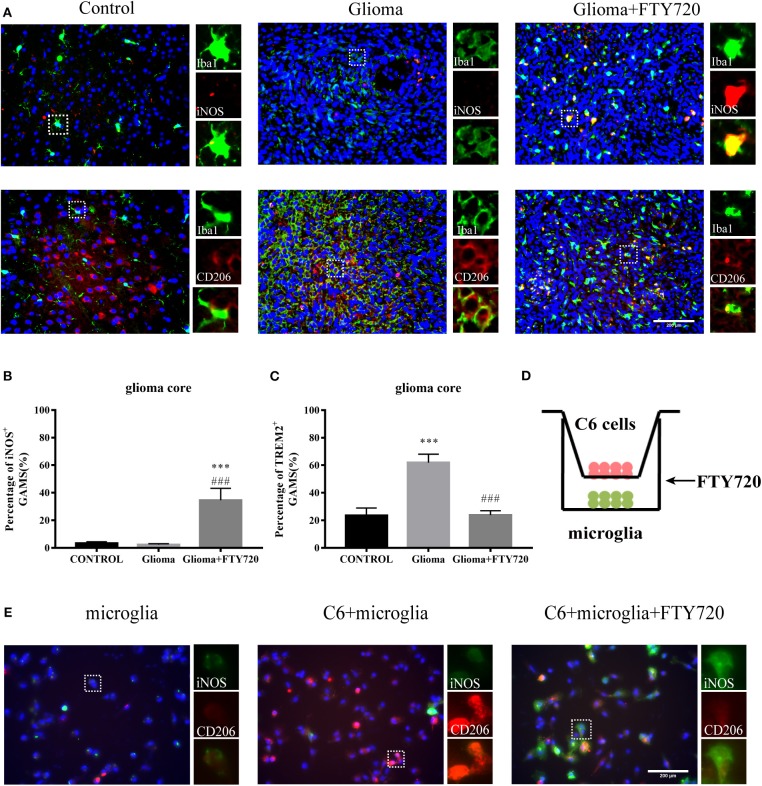
FTY720 impacts microglial polarization. **(A)** Immunofluorescent analysis of Iba1, iNOS, and CD206 expression in brain sections of C6-bearing rats, respectively. Left panel is 40× magnification and is amplified in the right panel (40×). *n* = 5–6, scale bar = 200 μm. **(B,C)** Representative analysis of iNOS-positive GAMs and CD206-positive GAMs in brain sections. Data are presented as mean ± SEMs, *n* = 5–6. ***p < 0.001 vs. Control, ^*###*^*p* < 0.001 vs. Glioma. **(D)** The coculture system of C6 cells and microglia. **(E)** IF analysis of microglia polarization using M1 marker iNOS (green) and M2 marker CD206 (red). *n* = 4, scale bar = 100 μm.

The cell-surface marker Iba1 is commonly used to label total GAMs, while iNOS and CD206 mark pro-tumor and anti-tumor phenotypes, respectively. In our results, we observed that the co-localization of Iba1 and iNOS increased and CD206 co-localization with Iba1 decreased in the glioma core of rats after FTY720 treatment (Figures 5A–C). Additionally, we found similar results in a glioma–microglia co-culture system (Figures 5D,E). These results suggested that FTY720 could push GAMs into anti-glioma phenotypes in the glioma microenvironment.

## Discussion

GBM is the most prevalent malignant primary adult brain tumor. Its poor prognosis is a product of the transformed cells acting in collusion with the tumor microenvironment (17). Glioma cells and diverse cellular players, ranging from peripherally derived immune cells to various specialized organ-resident cell types, come together to form a complex niche that ultimately promotes tumor progression, namely, the tumor microenvironment (6, 31, 32). Accumulating evidence demonstrate that new treatment approaches for glioblastoma may inhibit glioma growth through modulation of the immunosuppressive microenvironment. The majority of immune cells within brain tumor are microglia and macrophages, which comprise up to 30% of the tumor mass (8). Accordingly, we attempted to identify effective therapeutic strategies targeting GAMs in the glioma microenvironment (33–35), given their significant role in the tumor immunosuppressive milieu. Previous studies have indicated that FTY720 exerts an anti-tumor effect on glioblastoma (18, 22, 36). However, there are no data on the effects of FTY720 on the glioma microenvironment. In our study, we revealed a novel mechanism by which FTY720 regulates GAMs in the glioma niche and thereby provides anti-glioma effects.

GAMs infiltrate, and accumulate in and around glioma tissue, which is attributed to the recruitment of glioma. Many factors including different kinds of cytokines, chemokines, and receptors mediate GAMs chemoattraction (13). Several chemoattractant factors, including MIF–CD74, CCL2–CCR2, and CXCL12–CXCR4 pathways, have been recently demonstrated to be associated with GAMs recruitment (15, 16, 27, 37). Therefore, we investigated the effect of FTY720 on GAMs chemoattraction. We found that FTY720 increased CXCR4 internalization on GAMs, which was similar to the functions of FTY720 used for multiple myeloma treatment (26). In this study, we investigated the mechanism by which FTY720 regulates CXCR4-mediated chemoattraction of GAMs.

**Figure 6:**
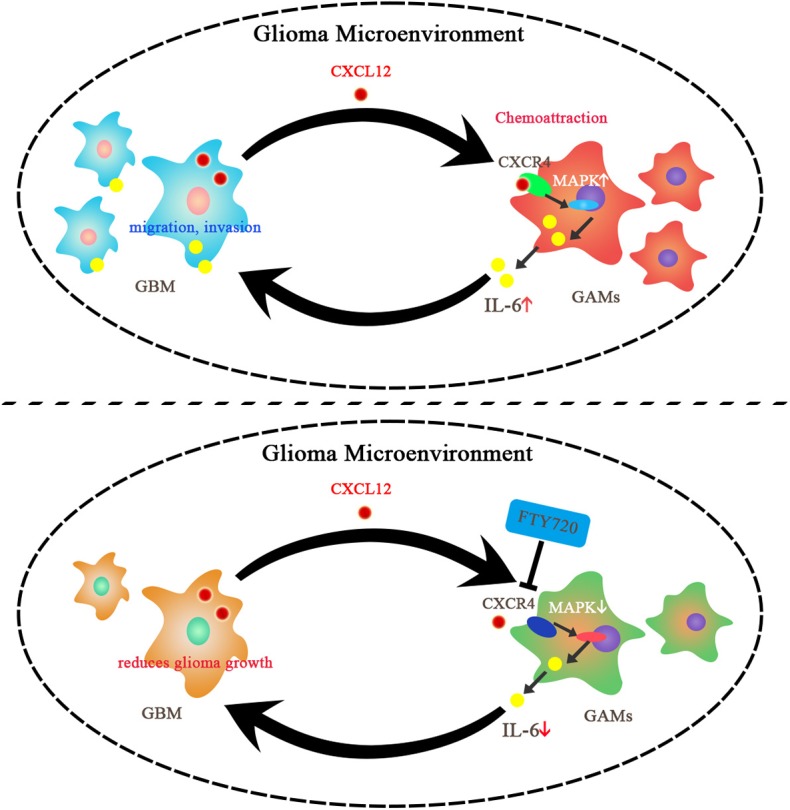
Summary of the impacts of FTY720 on glioma microenvironment. GBM can secrete CXCL12 to activate CXCR4 on the GAMs, which results in chemoattraction of GAMs and support for glioma growth. FTY720 treatment can induce CXCR4 internalization on GAMs, and thereby inhibit MAPK-mediated IL-6 secretion and suppress migration and invasion of glioma.

CXCR4, an important player in supportive interactions between tumor cells and other cells, participates in tumor cell proliferation, metastasis, angiogenesis, and the tumor microenvironment cross-talk in GBM. Studies have revealed that targeting the CXCR4 pathway may provide a therapeutic approach against glioma (12, 38). Our data suggested that FTY720 can block the cross-talk between glioma and GAMs by increasing microglial CXCR4 internalization rather than by affecting CXCL12 secretion by glioma. Next, we investigated the specific pathways downstream of CXCR4, which may be affected by FTY720. We found that FTY720 can inhibit the activation of ERK, P38, and JNK subsequent to CXCR4 internalization. We also observed that FTY720 reduced MAPK-mediated release of IL-6 from microglia. This observation is in line with other studies that have established a role for MAPKs in the production of inflammatory cytokines and chemokines, which contribute to glioma growth, migration, and invasion (13, 39, 40). Several studies have shown that IL-6 is a crucial cytokine in glioma development and is important for glioma proliferation, invasion, and differentiation (15, 28, 41, 42). Additionally, we observed inhibition of glioma migration and invasion following treatment with FTY720 in a co-culture system. It is therefore conceivable that FTY720 can target the glioma microenvironment by inhibiting microglial MAPK-mediated IL-6 secretion downstream of CXCR4 internalization. This potential molecular mechanism is illustrated in Figure 6.

It has been recognized that cancer immunotherapy may be more effective if the pro-tumor phenotype of GAMs can be converted to an anti-tumor phenotype. Therefore, we examined the phenotype of GAMs in the glioma microenvironment after FTY720 treatment. The results indicated that FTY720 could push GAMs to anti-glioma phenotypes, thereby inhibiting the growth of glioma.

## Data Availability Statement

The datasets generated for this study are available on request to the corresponding author.

## Ethics Statement

All the animal experiments were carried out in accordance with the approval of the Animal Research Committee of Nanjing Medical University (approval number: IACUC-1709016).

## Author Contributions

X-DG contributed to the conception and design, performing the experiments, acquisition, analysis, interpretation of data, drafting and revising the article, and final approval of the version to be published. JJ and T-FX performed the experiments, acquisition, analysis and interpretation of data, and final approval of the version to be published. Y-QS and R-BG performed the experiments. HC contributed to acquisition, analysis and interpretation of data. X-LS contributed to conception and design, acquisition, analysis and interpretation of data, revising the article critically for important intellectual content, and final approval of the version to be published.

### Conflict of Interest

The authors declare that the research was conducted in the absence of any commercial or financial relationships that could be construed as a potential conflict of interest.

## Abbreviations

FDA: Food and Drug Administration
GAMs: glioma-associated microglia and macrophages
GBM: glioblastoma
IACUC: Institutional Animal Care and Use Committee
MAPK: mitogen-activated protein kinase
MRI: magnetic resonance imaging
S1P: sphingosine-1-phosphate
S1PRs: sphingosine-1-phosphate receptors.
